# Mendelian Randomization Analysis of Systemic Iron Status and Risk of Metabolic Dysfunction-Associated Steatotic Liver Disease

**DOI:** 10.3390/metabo16060356

**Published:** 2026-05-25

**Authors:** Wuyang Yue, Yi Yang, Jinling Ma, Jiale Zhang, Xinhui Wang, Junxia Min, Fudi Wang

**Affiliations:** 1 The Second Affiliated Hospital, School of Public Health, State Key Laboratory of Experimental Hematology, Zhejiang University School of Medicine, Hangzhou 310058, China; wuyangyue@zju.edu.cn; 2 School of Public Health, Sir Run Run Shaw Hospital, Zhejiang University School of Medicine, Hangzhou 310058, China; yangyi0310@zju.edu.cn (Y.Y.); majinling0704@zju.edu.cn (J.M.); zhangjiale6@zju.edu.cn (J.Z.); 3 The First Affiliated Hospital, Institute of Translational Medicine, Zhejiang Key Laboratory of Frontier Medical Research on Cancer Metabolism, Zhejiang University School of Medicine, Hangzhou 310058, China; 4 Global Innovation Institute of Element Science (GIIES-JLU), The First Hospital of Jilin University, Changchun 130021, China

**Keywords:** iron, ferroptosis, Mendelian randomization, steatosis, fibrosis

## Abstract

**Highlights:**

**What are the main findings?**

- Genetically elevated systemic iron status is causally associated with increased risks of hepatic steatosis and fibrosis/cirrhosis in MASLD.

**What are the implications of the main findings?**

- Iron homeostasis and ferroptosis represent potential targets for risk stratification and therapeutic intervention in MASLD.

**Abstract:**

**Objective**: Metabolic dysfunction-associated steatotic liver disease (MASLD) is a global public health crisis, progressing to hepatic cirrhosis and hepatocellular carcinoma. This study investigated the causal role of systemic iron status in MASLD progression. **Methods**: A two-sample Mendelian randomization (MR) design was implemented, with genetic variants serving as instrumental variables for four core systemic iron biomarkers. Outcome data for hepatic steatosis (8785 cases; 912,105 controls) and hepatic fibrosis/cirrhosis (3798 cases; 904,599 controls) were extracted from the FinnGen and UK Biobank databases. Multiple complementary MR methodologies and three instrumental variable selection strategies were applied to ensure robust causal inference. **Results**: Genetically predicted higher serum iron (odds ratio, OR: 1.42, 95% confidence interval, 95% CI: 1.34, 1.50), ferritin (OR: 1.84, 95% CI: 1.55, 2.18), and transferrin saturation (TfSat, OR: 1.24, 95% CI: 1.19, 1.30), together with lower total iron-binding capacity (TIBC, OR: 0.81, 95% CI: 0.77, 0.85), were significantly associated with increased hepatic steatosis risk (*p* < 0.00625). Similar associations were observed for hepatic fibrosis/cirrhosis: serum iron (OR: 1.66, 95% CI: 1.29, 2.14), ferritin (OR: 2.52, 95% CI: 1.52, 4.18), TfSat (OR: 1.40, 95% CI: 1.19, 1.63), and reduced TIBC (OR: 0.70, 95% CI: 0.60, 0.81). MR-Bayesian model averaging prioritized serum iron (MIP: 0.85, ${\hat{\theta }}_{\mathrm{MACE}}$: 0.295; PP: 0.725; ${\hat{\theta }}_{\lambda }$: 0.344) as the top-ranked factors for steatosis and TIBC (MIP: 0.604, ${\hat{\theta }}_{\mathrm{MACE}}$: −0.240; PP: 0.476, ${\hat{\theta }}_{\lambda }$: −0.358) for fibrosis/cirrhosis. **Conclusions**: Elevated systemic iron status causally drives MASLD onset and progression, highlighting iron homeostasis and ferroptosis as potential targets for prevention and clinical management.

## 1. Introduction

MASLD has emerged as a major global public health challenge, affecting approximately one-third of the adult population worldwide. It represents the leading cause of chronic liver disease and contributes substantially to cirrhosis, hepatocellular carcinoma (HCC), and liver-related mortality [1,2]. Over the past decades, both disease prevalence and disability-adjusted life years attributable to MASLD have increased steadily, imposing a growing burden on healthcare systems and society [3].

The rising incidence of MASLD is associated with lifestyle and metabolic problems. Excessive calorie intake, obesity, insulin resistance and lack of exercise all push the liver toward damage over time. Thyroid hormones are key regulators of systemic lipid metabolism, controlling lipolysis, fatty acid oxidation, lipogenesis, and cholesterol metabolism [4]. Moreover, dietary patterns are major determinants of hepatic lipid metabolism and MASLD pathogenesis. A high-calorie, high-fat, high-fructose, and high-carbohydrate dietary pattern promotes hepatic lipid accumulation and metabolic imbalance. Dietary imbalance may additionally influence iron metabolism and contribute to disease progression [5].

Iron deficiency is defined as serum ferritin < 12 µg/L in individuals with infection or inflammation, and <30 µg/L in apparently healthy individuals. Risk of iron overload is defined as ferritin > 150 µg/L in females and >200 µg/L in males for apparently healthy individuals, and >500 µg/L for non-healthy individuals. The same cut-offs apply to adolescents aged 10 to less than 20 years and adults aged 20–59 years. For pregnant women, iron deficiency is defined as <15 µg/L in those with infection or inflammation and <70 µg/L in apparently healthy individuals. It should be noted that markers of inflammation should be assessed along with ferritin concentration, and ferritin adjusted as necessary [6]. In another study, the reference range for serum iron is 50–150 μg/dL (9.0–26.9 μM) for men and 35–145 μg/dL (6.3–26.0 μM) for women. TIBC was measured using a Hitachi 747–200 chemistry analyzer (Hitachi, Ltd., Tokyo, Japan), employing an automated method that determines unbound iron-binding capacity via the FerroZine–iron complex. TIBC is calculated as the sum of unbound iron-binding capacity and serum iron, with a reference range of 250–400 μg/dL (44.8–71.6 μM). TfSat was derived as (serum iron/TIBC) × 100%, with a reference range of 14–50%. Ferritin levels were measured on a Bayer ACS:180 analyzer (Bayer Corporation, Tarrytown, NY, USA) using a two-site chemiluminometric sandwich immunoassay. The ferritin reference range is 20–300 µg/L for men and 20–200 µg/L for women [7]. Daily iron intake originates from diet, water, and environmental exposure, with recommended intakes of 8 mg/day for males and 18 mg/day for females of childbearing age, with a tolerable upper limit of 45 mg/day [8]. The role of iron pathologies, which are among the most common micronutrient disorders, is increasingly recognized. Free ferrous iron promotes the conversion of superoxide anions and hydrogen peroxide into highly reactive hydroxyl radicals. Excessive hydroxyl radicals trigger intense oxidative stress, promote lipid peroxidation, and induce hepatocyte damage and ferroptosis. Therefore, this metal-catalyzed reaction represents an important mechanistic link between iron overload, oxidative stress, and the progression of MASLD and hepatic fibrosis [9]. Ferroptosis is an iron-dependent form of regulated cell death driven by the catastrophic accumulation of lipid peroxides on cellular membranes. It is primarily caused by the failure of the glutathione peroxidase 4 (GPX4)-dependent antioxidant system, which normally detoxifies phospholipid hydroperoxides [10]. In the context of MASLD, ferroptosis serves as a critical driver facilitating the transition from simple steatosis to metabolic dysfunction-associated steatohepatitis and progressive hepatic fibrosis [11]. Therefore, therapeutic strategies targeting ferroptosis—such as iron chelation, GPX4 activation, or antioxidant supplementation—hold promise for halting disease progression. Experimental studies consistently show that MASLD models exhibit hepatic iron accumulation and lipid peroxidation, along with dysregulation of ferroptosis-related pathways, supporting their role in disease pathogenesis [12].

Epidemiological studies further corroborate these findings. Serum ferritin concentrations are typically elevated in individuals with MASLD, and higher ferritin concentrations have been linked to increased disease severity and progression of fibrosis [13,14,15,16]. Studies based on large population databases have reported that serum iron levels may be inversely linked to the risk of MASLD, whereas ferritin levels tend to show a positive association [14,17]. In cohorts of patients with MASLD, elevated serum iron and transferrin saturation have also been associated with an increased risk of hepatocellular carcinoma and liver-related adverse outcomes [18]. Our previous study demonstrated that individuals with liver cirrhosis exhibit significantly lower concentrations of both serum transferrin and hepatic transferrin [19]. Collectively, these data highlight the key contribution of disrupted iron metabolism in the initiation and progression of MASLD. Several findings are vulnerable to residual confounding from metabolic comorbidities, reverse causation caused by liver injury altering iron metabolism, and measurement bias related to cross-sectional biomarker assessment. MR is a statistical method that leverages genetic variants as instrumental variables to assess causal relationships between exposures and outcomes, thereby minimizing these limitations. Recent genome-wide association studies (GWAS) have identified genetic variants associated with iron-related traits, providing robust instrumental variables for MR analyses [20].

**Figure 1:**
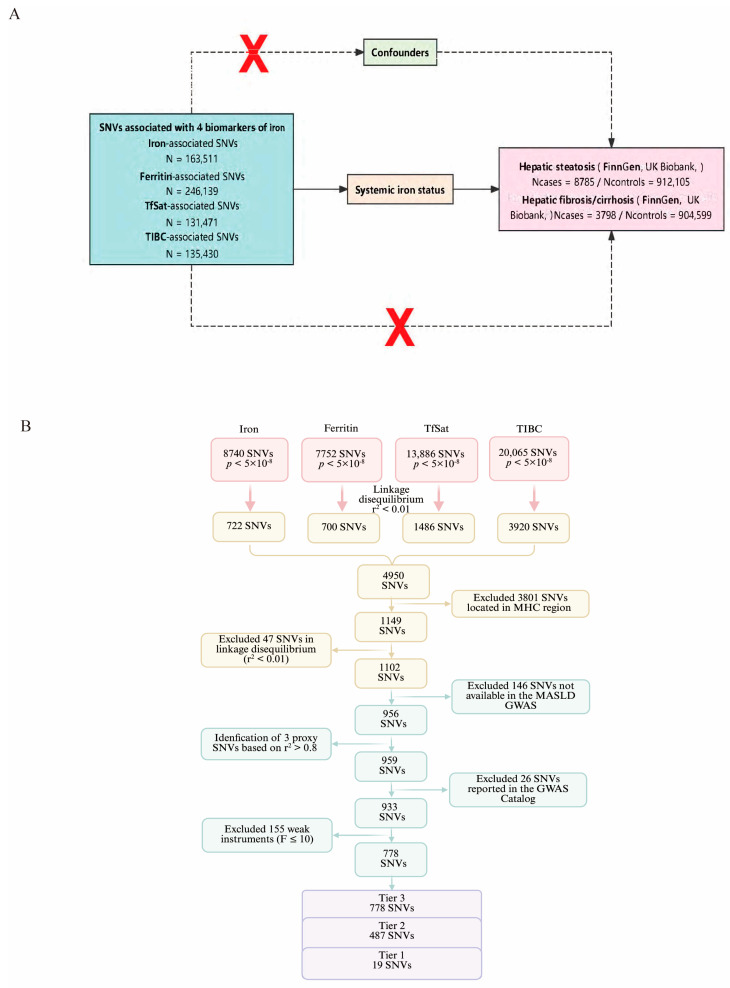
**Graphical overview of the two-sample MR study design.** (**A**) By using genetic instruments associated with these four iron status biomarkers, the MR approach can be used to estimate the causal effect of systemic iron status on the risk of hepatic steatosis and hepatic fibrosis/cirrhosis. The arrows in the figure denote the assumptions of MR analysis, indicating that instrumental variables correlate with exposure, independent of confounders, and affect outcome solely via exposure. Abbreviations: MR, Mendelian randomization; SNV, single-nucleotide variation; TIBC, total iron-binding capacity; TfSat, transferrin saturation. (**B**) Flowchart illustrating the selection and prioritization of instrumental SNVs for MR analyses of iron status biomarkers and MASLD. IVs were subsequently filtered through sequential quality-control procedures. Abbreviations: IV, instrumental variable; MR, Mendelian randomization; SNV, single-nucleotide variation; MASLD, metabolic dysfunction-associated steatotic liver disease.

To address these knowledge gaps, we performed a comprehensive two-sample MR analysis to systematically dissect the causal impact of four core iron status biomarkers (serum iron, ferritin, TfSat, and TIBC) on MASLD progression (Figure 1A). By integrating large-scale GWAS summary statistics from FinnGen and UK Biobank (encompassing 8785 hepatic steatosis cases/912,105 controls and 3798 hepatic fibrosis/cirrhosis cases/904,599 controls of European ancestry) [21], employing inverse-variance weighting (IVW), weighted median, MR–Egger regression, MR-PRESSO, and heterogeneity tests, and implementing three rigorous instrumental variable (IV) selection strategies (Figure 1B), our objective was to: (1) evaluate the causal role of systemic iron status in hepatic steatosis and fibrosis progression; (2) identify stage-specific iron biomarkers for MASLD risk stratification; and (3) provide robust genetic evidence to support iron homeostasis as a rational therapeutic target for MASLD prevention and clinical management. This study advances the field by resolving ambiguities from prior observational and limited MR studies, offering robust causal evidence into the role of iron metabolism in MASLD pathogenesis.

## 2. Methods

### 2.1. MR Analysis Study Design

Under the multiplicative random-effects IVW paradigm, genetic variations associated with all four iron status markers were used in the MR study. Alternative IV selection strategies and many complementary MR techniques, such as fixed-effect IVW, simple median, weighted median, MR–Egger, penalized weighted median, MRMix, and MR-BMA, were also used to assess the robustness of the results. Genetic variants (single-nucleotide variants, SNVs) were used as IVs. Three fundamental presumptions underpin the MR framework’s validity: the genetic variants must have a strong correlation with the exposure; they must be unaffected by potential confounders that could affect the exposure–outcome relationship; and they must only influence the outcomes through the exposure, not through other pathways. Figure 1 shows a schematic overview of the study design.

### 2.2. Data Sources from Three Genome-Wide Association Studies

Summary statistics for systemic iron status were obtained from a meta-analysis of three genome-wide association studies conducted in the UK, Iceland, and Denmark. These studies reported genetic associations with four iron-related biomarkers: serum iron (*n* = 163,511), ferritin (*n* = 246,139), transferrin saturation (TfSat; *n* = 131,471), and total iron-binding capacity (TIBC; *n* = 135,430) [22]. Detailed cohort characteristics are provided in Supplementary Table S1.

Genome-wide association summary statistics for hepatic steatosis and hepatic fibrosis/cirrhosis were obtained from large biobank resources including the FinnGen (R12) and UK Biobank databases. The hepatic steatosis dataset included 8785 cases of European ancestry and 912,105 controls, while the hepatic fibrosis/cirrhosis dataset contained 3798 cases and 904,599 controls of European ancestry [21]. Disease definitions were based on ICD-10 diagnostic codes: hepatic steatosis (ICD-10 K76.0) and hepatic fibrosis/cirrhosis (ICD-10 K74). Individuals with excessive or long-term alcohol intake were excluded during participant enrollment; detailed information is available in the References. Each release’s FinnGen disease endpoint definitions and corresponding controls may be found at https://www.finngen.fi/en/researchers/clinical-endpoints accessed on 22 October 2024 [21]. All datasets used in this study are publicly available, and the original studies received ethical approval with informed consent from participants.

### 2.3. Choosing the Instrumental Variables

A series of procedures for quality control was performed to identify valid instrumental SNVs. First, variants significantly associated with iron-related exposures at the genome-wide threshold (*p* < 5 × 10^−8^) were selected as candidate IVs. When the selected SNVs were not present in the outcome datasets, proxy variants were identified based on linkage disequilibrium utilizing the 1000 Genomes Project’s European reference panel (r^2^ > 0.8).

To ensure independence between variants, SNVs were clumped using the PLINK clustering procedure with an LD threshold of r^2^ < 0.01. Within each LD cluster exceeding this threshold, this study retained solely the SNV with the most significant association (lowest *p*-value). Potential associations with confounders were evaluated using the Pheno Scanner database (http://www.phenoscanner.medschl.cam.ac.uk, accessed on 28 November 2025), and variants associated with confounding traits or directly with the outcomes (*p* < 5 × 10^−8^) were excluded [23]. SNVs within the MHC region on chromosome 6 were excluded due to its intricate linkage disequilibrium structure. Genomic positions were defined according to Genome Reference Consortium Human Build 37 (hg19) [24].

The IVs were chosen using three different approaches. SNVs were to be concurrently associated with each of the four iron-related statuses in Tier 1. In Tier 2, SNVs had to show directionally consistent impacts on systemic iron status across the remaining biomarkers and be associated with at least one of the four biomarkers. Tier 3 included all SNVs associated with any of the iron-related biomarkers. Tier 1 was used for the primary analysis since systemic iron status was thought of as an integrated phenotype rather than a collection of distinct indicators. Tiers 2 and 3 were used to further evaluate the primary findings’ robustness.

### 2.4. Statistical Analysis

The relationship between systemic iron levels and hepatic steatosis, as well as hepatic fibrosis/cirrhosis, was assessed through several MR techniques. Odds ratios (ORs) were determined for every one standard deviation rise in genetically inferred iron status. The main analysis employed the multiplicative random-effects IVW method, known for its ability to generate reliable estimates when multiple instrumental variables are used [25].

Additional MR methods were applied to assess the robustness of the findings, including fixed-effect IVW, weighted median [26], simple median, penalized weighted median, and MR–Egger regression [27]. To further account for and reduce potential bias induced by horizontal pleiotropy, a supplementary mixture-model-based Mendelian randomization method, which is MRMix, was applied in the present analysis [28].

MR-BMA was implemented to identify and prioritize the most likely causal iron biomarkers [29]. This approach evaluates all possible combinations of exposures and calculates posterior probabilities (PPs) for each model. From these probabilities, marginal inclusion probabilities (MIPs) are derived to quantify the likelihood that a specific biomarker represents a true causal risk factor. Model-averaged causal estimates (${\hat{\theta }}_{\mathrm{MACE}}$ and estimated causal effect of risk factor λ (${\hat{\theta }}_{\lambda }$) were also calculated.

### 2.5. Sensitivity Analysis and Instrument Strength

The strength of each instrumental variable was evaluated using the F-statistic, which was computed based on the percentage of exposure variance explained by the genetic variations (R^2^), the number of instruments (k), and the sample size (*n*) [30]. The following formula can be used to get the F-statistic:


$$F=(\frac{R^{2}}{k})/(\frac{\left[1-R^{2}\right]}{\left[n-k-1\right]})$$

Weak instrument bias was indicated by an F-statistic less than 10. Lastly, Burgess’s design-based power calculation method was used to evaluate the statistical power of the Mendelian randomization analysis [31].

Cochran’s Q statistic was used to measure heterogeneity among IVs, and the intercept from MR–Egger regression was used to examine potential horizontal pleiotropy [32]. Outlying or influential variants were identified using Cook’s distance (Cd) and Cochran’s Q statistic. SNVs that had a Cd value higher than the relevant F-distribution’s median or a Q statistic larger than 10 were not included in the analysis [31].

Bonferroni correction was used to account for multiple testing, and the significance threshold was set at *p* < 0.00625 (0.05/8, corresponding to four exposures and two outcomes), while *p*-values between 0.00625 and 0.05 were thought to be suggestive.

All analyses were conducted in R (version 4.5.1; R Foundation for Statistical Computing) using the Two Sample MR (version 0.6.22) and MRMix packages (version 0.1.0).

## 3. Results

### 3.1. Features of SNVs Utilized as Genetic Tools

This study identified SNV sets associated with elevated systemic iron status (represented by serum iron, ferritin, and TfSat) and reduced systemic iron status (represented by TIBC) from the deCODE meta-analysis (Figure 1B). Supplementary Table S1 lists the impact on iron levels for each copy of the instrument SNV effect allele, represented as the number of SDs from the mean. Thus, after significance threshold screening, LD clumping, proxy selection, and exclusion of known pleiotropic variants, Strategy 1 yielded 19 independent SNVs that were used as the Tier 1 IV set (Supplementary Table S2). All employed IVs were sufficiently strong, with F-statistics exceeding 10 across Strategies 1, 2, and 3. The specific R^2^ values and F values are provided in Supplementary Tables S5–S7. According to post hoc power estimates, the study’s sample size was adequate for both hepatic steatosis (Supplementary Table S8) and hepatic fibrosis/cirrhosis (Supplementary Table S9).

### 3.2. Main Analysis

**Figure 2:**
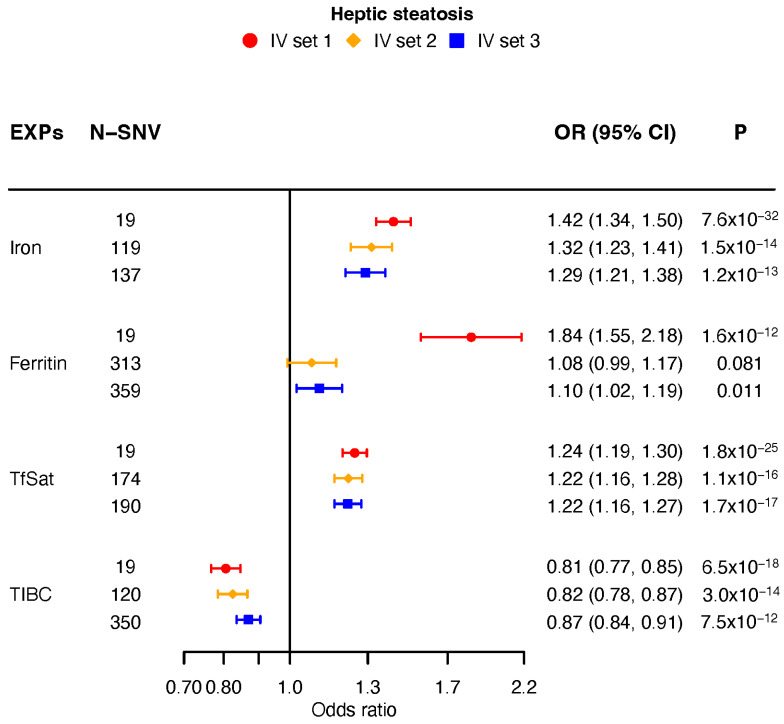
**Causal effects of systemic iron status on hepatic steatosis estimated by univariable MR using the multiplicative random-effects IVW method across three instrumental variable selection strategies.** Estimated causal effects of serum iron, ferritin, TfSat, and TIBC on hepatic fibrosis/cirrhosis risk (OR). The solid spots represent the estimates of the causal effects and the horizontal lines indicate the 95% CIs. The strategies for the 3 IV sets are indicated as red circle, orange diamond, and blue square, respectively. Abbreviations: 95% CI, 95% confidence interval; IVW, inverse-variance weighting; MR, Mendelian randomization; OR, odds ratio; SNV, single-nucleotide variation; TIBC, total iron-binding capacity; TfSat, transferrin saturation.

Overall, elevated iron, ferritin, and TfSat levels were associated with an elevated risk of liver injury mediated by genetic susceptibility to higher iron status; this finding was further supported by lower TIBC. The directional consistency and significance of differences for 19 separate SNVs in hepatic steatosis were assessed in accordance with Tier 1. Iron (OR: 1.42; 95% CI: 1.34, 1.50; *p* < 0.00625), ferritin (OR: 1.84; 95% CI: 1.55, 2.18; *p* < 0.00625), and transferrin saturation (TfSat; OR: 1.24; 95% CI: 1.19, 1.30; *p* < 0.00625) all showed a significantly elevated systemic iron status. On the other hand, total iron-binding capacity (TIBC; OR: 0.81; 95% CI: 0.77, 0.85; *p* < 0.00625) showed that individuals with lower systemic iron status exhibited a decreased risk (Figure 2, Supplementary Figure S1).

**Figure 3:**
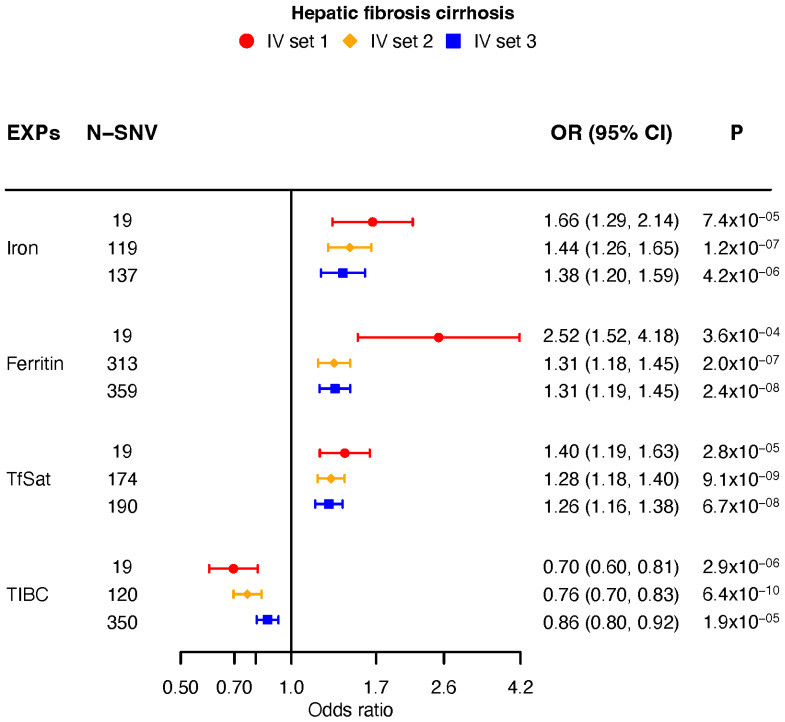
**Causal effects of systemic iron status on hepatic fibrosis/cirrhosis estimated by univariable MR using the multiplicative random-effects IVW method across three instrumental variable selection strategies.** Estimated causal effects of serum iron, ferritin, TfSat, and TIBC on hepatic fibrosis/cirrhosis risk (OR). The solid spots represent the estimates of the causal effects and the horizontal lines indicate the 95% CIs. The strategies for the three IV sets are indicated as red circle, orange diamond, and blue square, respectively. Abbreviations: 95% CI, 95% confidence interval; IVW, inverse-variance weighting; MR, Mendelian randomization; OR, odds ratio; SNV, single-nucleotide variation; TIBC, total iron-binding capacity; TfSat, transferrin saturation.

The findings showed a substantial causal relationship between an increased risk of hepatic fibrosis/cirrhosis and all four measures of elevated systemic iron status: iron (OR: 1.66; 95% CI: 1.29, 2.14; *p* < 0.00625), ferritin (OR: 2.52; 95% CI: 1.52, 4.18; *p* < 0.00625), TfSat (OR:1.40; 95% CI: 1.19, 1.63; *p* < 0.00625), and TIBC (OR: 0.70; 95% CI: 0.60, 0.81; *p* < 0.00625). Our findings support that increased systemic iron status is causally associated with a higher risk of hepatic steatosis and progression to hepatic fibrosis/cirrhosis (Figure 3, Supplementary Figure S2).

### 3.3. Sensitivity Analysis

This study used the gwasrapidd package in R to programmatically screen each SNV in the GWAS Catalog in order to reduce horizontal pleiotropy. In 29 November 2025, the catalog queries were executed. Substantial genome-wide associations were identified in domains that may represent alternative pathways contributing to the risk of hepatic fibrosis/cirrhosis and hepatic steatosis. The associated rsID variants detected by this screening were merged into an exclusion list and removed from the MR analysis set. Additionally, a variable was eliminated prior to MR estimation if an SNV showed genome-wide significance for the outcomes including hepatic steatosis and hepatic fibrosis/cirrhosis. For the remaining instruments, this study kept the catalog notes to help with interpretation of findings. To uncover potential alternative biological pathways and support variant filtering, prior genome-wide significant associations for each SNV and eligible proxy were retrieved from the GWAS Catalog; comprehensive cross-references are included in Supplementary Tables S10–S12. Hepatic steatosis and hepatic fibrosis/cirrhosis did not exhibit horizontal pleiotropy in terms of the MR–Egger regression intercept (*p* > 0.05) (Supplementary Tables S13 and S14). Lastly, in hepatic steatosis and hepatic fibrosis/cirrhosis, the Cochran’s Q statistics demonstrated that there was no heterogeneity between the assessed IV values for each biomarker when using the MR–Egger and IVW approaches (Supplementary Tables S15 and S16).

### 3.4. Analysis Using Different IV Selection Strategies

Increased systemic iron status was linked to a greater propensity for hepatic steatosis, in accordance with Tier 2 of the multiplicative random-effects IVW technique. In particular, TIBC was inversely correlated (OR: 0.82; 95% CI: 0.78, 0.87; *p* < 0.00625), whereas serum iron (OR: 1.32; 95% CI: 1.23, 1.41; *p* < 0.00625) and TfSat (OR: 1.22; 95% CI: 1.16, 1.28; *p* < 0.00625) were positively correlated. A positive but non-significant correlation was found with ferritin (OR: 1.08; 95% CI: 0.99, 1.17; *p* = 0.081) (Figure 2).

A higher risk of hepatic fibrosis/cirrhosis was linked to increasing systemic iron status. Significant positive associations were observed for serum iron (OR: 1.44; 95% CI: 1.26, 1.65; *p* < 0.00625), ferritin (OR: 1.31; 95% CI: 1.18, 1.45; *p* < 0.00625), and TfSat (OR: 1.28; 95% CI: 1.18, 1.40; *p* < 0.00625), whereas TIBC remained inversely associated (OR: 0.76; 95% CI: 0.70, 0.83; *p* < 0.00625) (Figure 3).

Higher systemic iron levels was associated with higher chances of both liver diseases in Tier 1, with significant associations with serum iron (OR: 1.42; 95% CI: 1.34, 1.50; *p* < 0.00625), ferritin (OR: 1.84; 95% CI: 1.55, 2.18; *p* < 0.00625), TfSat (OR: 1.24; 95% CI: 1.19, 1.30; *p* < 0.00625), and TIBC (OR: 0.81; 95% CI: 0.77, 0.85; *p* < 0.00625).

MASH was also substantially correlated with all four markers. TIBC demonstrated a protective effect (OR: 0.70; 95% CI: 0.60, 0.81; *p* < 0.00625), whereas elevated serum iron (OR: 1.66; 95% CI: 1.29, 2.14; *p* < 0.00625), ferritin (OR: 2.52; 95% CI: 1.52, 4.18; *p* < 0.00625), and TfSat (OR: 1.40; 95% CI: 1.19, 1.63; *p* < 0.00625) were associated with increased risk.

Increased systemic iron status is strongly correlated with the risks of hepatic steatosis and hepatic fibrosis/cirrhosis, according to results that consistently supported the primary analysis across three analytical methodologies.

Genetically predicted higher systemic iron status was linked to an increased risk of outcomes related to liver injury in Mendelian randomization analysis. Elevated serum iron, ferritin, and TfSat were substantially linked to an increased risk of hepatic steatosis in the primary analysis utilizing six different SNVs, but higher TIBC was linked to a lower risk. Overall, both hepatic steatosis and hepatic fibrosis/cirrhosis showed statistically significant associations with systemic iron status. Sensitivity tests using various Mendelian randomization techniques showed that there was no indication of horizontal pleiotropy and that effect directions were consistent. The robustness of the primary findings was supported by the generally similar results obtained from alternative instrumental variable selection procedures.

### 3.5. Analysis with Different MR Methods

This study conducted univariable MR analyses employing IVW, simple median, weighted median, MR–Egger, and penalized weighted median methodologies across three strategies. In Tier 1 for hepatic steatosis, all methods generally demonstrated a significant level of directional agreement with the results from the multiplicative random-effects IVW model (Supplementary Tables S17 and S18). For hepatic steatosis and hepatic fibrosis/cirrhosis, all other methods stayed true to the multiplicative random-effects IVW estimates in terms of direction.

This study used the MRMix method to look for and take into account possible horizontal pleiotropy. The estimated causal effects (θ) for serum iron, ferritin, TfSat, and TIBC were 0.45, 0.2, 0.02, and −0.15 for hepatic steatosis, and 0.46, 0.6, 0.26, and −0.31 for hepatic fibrosis/cirrhosis, respectively (Table 1).

Additionally, the non-linear MR-BMA method was utilized with IVs chosen from Tier 1 to rank the best models for hepatic steatosis and hepatic fibrosis/cirrhosis. The highest-ranked iron biomarkers for hepatic steatosis were iron (MIP: 0.85; $\hat{\theta }$_MACE_: 0.295), TIBC (MIP: 0.067; $\hat{\theta }$_MACE_: 0.005) and ferritin (MIP: 0.057; $\hat{\theta }$_MACE_: 0.005). These biomarkers were also used in the best models for PP > 0.02 (Table 2). The highest-ranked iron biomarkers for hepatic fibrosis/cirrhosis were TIBC (MIP: 0.604; $\hat{\theta }$_MACE_: −0.24), TfSat (MIP: 0.235; $\hat{\theta }$_MACE_: 0.051), Iron (MIP: 0.212; $\hat{\theta }$_MACE_: 0.049), and ferritin (MIP: 0.125; $\hat{\theta }$_MACE_: 0.057) (Table 3).

## 4. Discussion

In this large-scale MR study involving populations from Finland and the UK, a two-sample MR design was employed to investigate the role of genetically regulated iron metabolism in the risk of hepatic steatosis and hepatic fibrosis/cirrhosis. Our findings revealed strong associations between genetically predicted hepatic iron levels and an elevated risk of hepatic steatosis. Additionally, across multiple datasets, genetically predicted MASLD was robustly associated with higher levels of serum ferritin, iron, and TfSat.

Systemic iron homeostasis plays a key role in the pathogenesis of metabolic liver diseases, and our MR study provides genetic evidence supporting a causal role of elevated systemic iron status in both hepatic steatosis and hepatic fibrosis/cirrhosis. These findings align with and extend prior epidemiological and experimental evidence, while addressing critical limitations of observational research through genetic instrumental variable analysis.

Furthermore, three IV selection strategies (Tier 1/2/3) were integrated with multiple MR methods, including multiplicative random-effects IVW, fixed-effects IVW, median-based methods (simple median and weighted median), MR–Egger, MRMix, and MR-BMA. This comprehensive approach simultaneously controlled for horizontal pleiotropy and prioritized the ranking of iron biomarkers. The strengths of this study also include an increased number and strength of IVs, coupled with Bonferroni correction for multiple testing (*p* < 0.00625, accounting for four exposures and two outcomes), which effectively reduced the risk of false positives and improved the credibility of results. The directions of effect for different iron biomarkers were further clarified—for instance, decreased TIBC was associated with a higher risk of both hepatic steatosis and hepatic fibrosis/cirrhosis—providing precise references for the clinical application of these biomarkers. In contrast, the referenced literature did not implement multiple-testing correction, leading to more generalized and less precise interpretations of their findings. Among all iron biomarkers, ferritin exhibited the highest θ value (0.60) for hepatic fibrosis/cirrhosis, suggesting that its potential pathogenic role in liver fibrosis may have been underestimated by the IVW method. It has been shown that iron has a protective effect on kidney diseases in the general population [33]. Previous MR studies have demonstrated that elevated iron status independently increases the risk of cardioembolic ischemic stroke, with this association being partially mediated by diastolic blood pressure. These findings support iron status as a potentially modifiable cardiovascular risk factor and highlight the need for further mechanistic investigation and evaluation of iron-targeted interventions for stroke prevention [34,35]. In addition, previous MR studies have suggested that higher iron status may reduce the risk of coronary artery disease (CAD). These findings may highlight a therapeutic target [36]; however, some studies found higher iron status shows a protective trend against coronary heart disease [37]. In addition, higher levels of iron status, although protective for coronary artery disease, can have detrimental effects regarding type 2 diabetes mellitus (T2DM) [38]. Our team previously conducted MR studies investigating the associations between iron status and both diabetes mellitus and IgA nephropathy [39]. Abnormal iron biomarkers are also closely linked to neuropsychiatric disorders: genetically predicted serum iron, ferritin and transferrin saturation are positively associated with depression and psychogenic disorders. In females, iron overload may reduce the risk of certain premenstrual symptoms [40]. In oncology, elevated ferritin, serum iron and transferrin saturation have been causally associated with increased risks of ovarian, liver and cervical cancers [41]. Higher iron status is positively associated with improved forced expiratory volume in 1 s (FEV1) and forced vital capacity (FVC) lung function, while genetic iron excess also elevates the susceptibility to gout [42]. Collectively, these findings indicate that systemic iron homeostasis is broadly involved in the pathogenesis of cardiovascular, neuropsychiatric, reproductive, malignant, pulmonary, and metabolic disorders. This evidence further supports the present findings that dysregulated iron metabolism plays a causal role in the development and progression of MASLD and hepatic fibrosis. The current study further extends our research framework by exploring the causal link between iron and fatty liver disease. Notably, our study is distinct from two published MR studies [43,44]. Under the multiplicative random-effects IVW paradigm, genetic instruments linked to all four iron biomarkers were used in the MR study. Alternative IV selection strategies and many complementary MR techniques were also used to assess the robustness of the results. Moreover, compared with previous studies, our study included the largest sample size and demonstrated associations between lower transferrin levels and/or higher transferrin saturation with hepatic fibrosis/cirrhosis. This is the first finding that all four iron biomarkers are consistent with hepatic fibrosis/cirrhosis. These findings further support iron homeostasis as not only a causal risk factor for hepatic steatosis but also a contributor for liver fibrosis.

Iron is an essential micronutrient that plays critical roles in cellular respiration, DNA synthesis, and cellular proliferation [45]. Iron plays a crucial catalytic role in the Haber–Weiss reaction. Under physiological conditions, iron catalyzes reactive oxygen species generation through the coupled Haber–Weiss and Fenton reactions. In this process, superoxide anion (O_2_^•−^) reduces ferric iron (Fe^3+^) to ferrous iron (Fe^2+^), while Fe^2+^ subsequently reacts with hydrogen peroxide (H_2_O_2_) via the Fenton reaction to generate highly reactive hydroxyl radicals (•OH). The overall Haber–Weiss cycle therefore amplifies oxidative stress through continuous Fe^2+^/Fe^3+^ redox cycling. Excessive hydroxyl radical production promotes lipid peroxidation, mitochondrial dysfunction, hepatocyte injury, inflammatory activation, and ferroptosis, thereby contributing to the development and progression of MASLD and hepatic fibrosis. Ferritin exhibits strong physiological stability and high detection reliabilityand is widely used as a core marker of body iron reserves. Functionally, ferritin not only maintains intracellular iron homeostasis but also participates in the regulation of oxidative stress and inflammatory responses. Abnormal ferritin elevation is strongly associated with the occurrence and progression of MASLD, serving as a useful clinical indicator for disease risk stratification [46]. Numerous studies have emphasized the crucial role of iron in ferroptosis, highlighting the crucial key regulatory factors such as HDAC3, ACSL4, and cMyc. Dipyridamole exerts a favorable therapeutic effect on liver and kidney injury. Additionally, SLC7A11 deletion specifically facilitates ferroptosis onset under high-iron conditions. Mechanistically, excess iron promotes the generation of reactive oxygen species (ROS) and lipid peroxidation. Iron overload can activate hepatic stellate cells through oxidative stress and inflammatory signaling. This activation promotes collagen synthesis and fibrogenic pathways, which contribute to the development of liver fibrosis [47,48]. Ferroptosis, an iron-dependent type of controlled cell death that exacerbates inflammation and hepatocellular damage, can be triggered by this process [49]. In mouse models of MASLD, special diets induce hepatic iron accumulation, lipid peroxidation, and downregulation of ferroptosis-related genes, directly linking iron dysregulation to steatotic liver disease pathogenesis [50]. However, the role of ferroptosis remains unclear, underscoring the need for additional research to clarify its underlying processes in MASH [51,52]. With the advancement of clinical trials involving FOT1 and other novel iron chelators, these findings lay the foundation for identifying patient subgroups that may benefit from iron chelation therapy and for optimizing corresponding treatment strategies.

**Figure 4:**
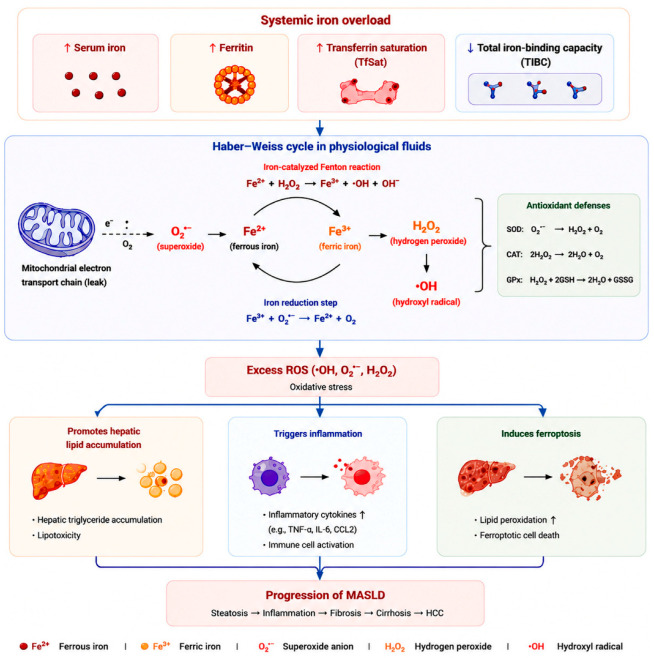
**Mechanistic pathways linking systemic iron overload to the progression of MASLD.** Elevated systemic iron status (higher serum iron, ferritin, and transferrin saturation, and lower total iron-binding capacity) enhances reactive oxygen species (ROS) generation via the iron-catalyzed Haber–Weiss cycle in physiological fluids. Excess ROS lead to oxidative stress, which promotes MASLD progression through three converging pathways: (1) hepatic lipid accumulation, (2) inflammation, and (3) ferroptosis, ultimately driving the progression from steatosis to inflammation, fibrosis, cirrhosis, and hepatocellular carcinoma (HCC).

In line with our Mendelian randomization findings, systemic iron overload can drive MASLD progression through multiple interrelated pathways. Elevated iron enhances ROS production via the Haber–Weiss and Fenton reactions, leading to oxidative stress that triggers hepatocyte lipid accumulation [9]. Iron-induced oxidative damage also activates inflammatory pathways, including cytokine release (e.g., TNF-α, IL-6, CCL2) and immune cell activation, further exacerbating liver injury [46]. Concurrently, iron-dependent lipid peroxidation induces ferroptosis, a regulated cell death process, amplifying hepatocellular damage [50]. Collectively, these mechanistic pathways—oxidative-stress-mediated lipid accumulation, inflammation, and ferroptosis—provide a molecular link between iron dysregulation and MASLD progression, highlighting iron homeostasis and ferroptosis as potential therapeutic targets (Figure 4).

In this study, genetically predicted increases in serum iron, ferritin, and TfSat were significantly associated with a higher risk of hepatic steatosis. In contrast, lower TIBC, which reflects greater iron reserves, was associated with increased disease risk. These results are consistent with observational studies that have shown that hepatic steatosis patients usually have elevated serum ferritin and iron levels, alongside decreased TIBC [53,54]. Our findings provide genetic evidence that iron overload is causally associated with MASLD. Previous observational studies have reported significantly elevated levels of serum iron and ferritin, along with decreased TIBC, in MASLD patients. By leveraging MR analysis to eliminate confounding factors, the present study is the first to confirm a causal relationship between systemic iron status and MASLD.

Prior studies mainly focused on the relationship between iron status and hepatic steatosis, without clearly defining its role in fibrosis progression [55]. Our MR-BMA analysis prioritized iron (MIP: 0.85) as the core iron biomarker for hepatic steatosis, followed by TfSat (MIP: 0.187) and TIBC (MIP: 0.067). In contrast, the θ value for TIBC was negative (−0.15), indicating that lower TIBC—an indicator of increased systemic iron reserves—is associated with a higher risk of hepatic steatosis. This stage-specific biomarker ranking aligns with clinical observations that ferritin reflects both iron stores and inflammatory status, making it a sensitive marker of liver injury in hepatic steatosis. Our study established that elevated peripheral iron-related traits are causal risk factor for hepatic fibrosis/cirrhosis. Among the iron biomarkers, ferritin showed the strongest effect in the primary analysis. This finding helps address an important gap in the literature.

In clinical practice, our MR analyses help eliminate confounding biases and provide genetic evidence for the association between iron status and liver disease, offering up-to-date and comprehensive insights into their causal relationship. These findings underscore the robustness and reliability of our results, providing strong support for causal inference. Elevated systemic iron status was associated with an increased risk of both hepatic steatosis and hepatic fibrosis/cirrhosis, although the direction and statistical significance of these associations varied across different iron biomarkers. Specifically, for hepatic steatosis, higher levels of serum iron, ferritin, and TfSat were significantly associated with an elevated disease risk, whereas a reduced TIBC was also associated with a significantly increased risk of hepatic steatosis. Regarding hepatic fibrosis/cirrhosis, elevated serum iron and TfSat levels showed a tendency toward increased risk, while decreased TIBC was significantly associated with a higher risk of hepatic fibrosis/cirrhosis.

Beyond mechanistic insights, our findings have important implications for nutritional practice and lifestyle modification. This Mendelian randomization analysis demonstrates that genetically predicted elevations in systemic iron status—particularly serum iron, ferritin, and transferrin saturation—are causally associated with increased risks of hepatic steatosis and advanced fibrosis. As genetic instruments reflect lifelong differences in iron exposure, these results suggest that chronic iron excess may act as a modifiable environmental risk factor interacting with dietary habits. From a nutritional perspective, high intake of bioavailable iron—such as heme iron from red and processed meats, iron-fortified foods, and long-term supplementation—may exacerbate metabolic liver injury in susceptible individuals. While adequate iron intake remains essential, excessive exposure may promote hepatic lipid accumulation, oxidative stress, and ferroptosis-driven damage, particularly in metabolically vulnerable populations.

Importantly, these findings provide a framework for translating genetic evidence into precision dietary strategies. Individuals at high risk of MASLD, including those with obesity, insulin resistance, or type 2 diabetes, may benefit from personalized nutritional guidance aimed at avoiding unnecessary iron overload. Practical strategies include cautious use of iron supplementation, moderation of red meat intake, and adherence to dietary patterns rich in plant-based foods, fiber, and antioxidants. Moreover, integrating genetic risk prediction with nutritional assessment may help identify individuals with heightened sensitivity to iron exposure, enabling targeted interventions, biomarker monitoring, and prevention strategies that prioritize optimal iron homeostasis rather than excessive intake.

Nevertheless, our study also has several limitations. First, the analysis was based solely on summary-level GWAS data, which prevented stratified analyses by factors such as age, sex, or drinking status, potentially hiding variations across different subgroups. Second, the exposure–response relationship between iron homeostasis and the severity of hepatic steatosis or hepatic fibrosis/cirrhosis—such as the degree of hepatic steatosis or the stage of hepatic fibrosis/cirrhosis—was not examined; the study only focused on the presence or absence of disease risk, not the severity of that risk.

Using comprehensive MR analyses with multiple methods and strategies, this study provides strong evidence that higher systemic iron levels are a causal risk factor for hepatic steatosis and are significantly associated with MASLD. Important iron metabolism biomarkers—including TfSat, serum iron, and TIBC—serve as key contributors of these associations. These findings provide valuable genetic insights into the underlying causes of hepatic steatosis and hepatic fibrosis/cirrhosis, offer new perspectives for clinical screening, risk assessment, and targeted treatment, and point to important directions for future research on iron-related liver diseases. Further studies in larger populations are warranted. Our future studies will be conducted in larger populations. Meanwhile, clinical intervention studies will be performed in patients with hepatic fibrosis and cirrhosis, using TfSat and ferritin as stage-specific targets.

## 5. Conclusions

In conclusion, this rigorously designed two-sample Mendelian randomization study provides strong genetic evidence that systemic iron homeostasis dysregulation is a causal driver of MASLD development and progression, with ferroptosis as a key mechanistic mediator linking iron overload to hepatocellular injury and fibrogenesis. Through systematic interrogation of four core iron biomarkers (serum iron, ferritin, TfSat, and TIBC) across the disease continuum from early hepatic steatosis to advanced fibrosis/cirrhosis, we leveraged GWAS encompassing 8785 steatosis cases with 912,105 controls and 3798 fibrosis/cirrhosis cases with 904,599 controls. Integration of three instrumental variable selection strategies, multiple robust MR and comprehensive sensitivity analyses confirmed consistent causal associations between elevated serum iron, ferritin, and TfSat, decreased TIBC, and an increased risk of hepatic steatosis, with all associations achieving statistical significance at *p* < 0.00625 after Bonferroni correction. MR-BMA prioritized iron and ferritin as top causal factors, with ferritin exerting the most pronounced effect on advanced fibrosis/cirrhosis (OR: 2.52, 95% CI: 1.52, 4.18, *p* < 0.00625). These findings resolve longstanding ambiguities from observational studies, identify stage-specific biomarkers for risk stratification and establish iron homeostasis and ferroptosis as synergistic rational therapeutic targets, offering critical insights to inform precision prevention and clinical management strategies for mitigating the global public health burden of metabolic dysfunction-associated steatotic liver disease. In summary, our findings highlight the interplay between systemic iron status and ferroptosis in the pathogenesis of MASLD.

## Figures and Tables

**Table 1:** Associations between genetically instrumented systemic iron status and hepatic steatosis and hepatic fibrosis/cirrhosis using the 6 single-nucleotide variations associated with all 4 iron biomarkers, respectively.

Exposure	Hepatic Steatosis—MRMix			Hepatic Fibrosis/Cirrhosis—MRMix		
	θ	π_0_	σ^2^	θ	π_0_	σ^2^
Iron	0.45	0.999	1.47 × 10^−3^	0.46	0.999	5.48 × 10^−3^
Ferritin	0.20	0.999	1.59 × 10^−2^	0.60	0.999	4.24 × 10^−2^
TfSat	0.02	0.923	8.26 × 10^−4^	0.26	0.999	2.80 × 10^−3^
TIBC	−0.15	0.999	1.92 × 10^−2^	−0.31	0.999	4.07 × 10^−2^

Data source and sample size: A case–control GWAS of hepatic steatosis in individuals of European ancestry (*n* = 8785 and 912,105, respectively) was obtained from FinnGen and the UK Biobank study. A separate case–control GWAS of hepatic fibrosis/cirrhosishepatic fibrosis/cirrhosis of European ancestry (*n* = 3798 and 904,599, respectively) was also derived from FinnGen and the UK Biobank study. Genetic instruments were selected based on three genome-wide association studies of blood iron biomarkers from Iceland, the UK, and Denmark, including blood levels of ferritin (*n* = 246,139), TIBC (*n* = 135,430), iron (*n* = 163,511) and TfSat (*n* = 131,471). Six SNVs associated with all four iron status biomarkers at genome-wide significance for hepatic steatosis and hepatic fibrosis/cirrhosis (*p* < 5 × 10^−8^) were selected as genetic instruments for systemic iron status. θ, the estimates of causal effects generated by MRMix approach; π_0_, the proportion of valid instrumental variables; and σ^2^, the unknown variance parameter associated with the invalid instrumental variables. Abbreviations: MRMix, MR analysis using mixture model; TIBC, total iron-binding capacity; TfSat, transferrin saturation.

**Table 2:** Ranking of risk factors and models (sets of risk factors) for hepatic steatosis.

**(A) Model averaging for risk factors**			
**Ranking by MIP**	**Risk factor**	**MIP**	$\hat{\theta }$ **_MACE_**
1	Iron	0.850	0.295
2	TfSat	0.187	0.035
3	TIBC	0.067	0.005
4	Ferritin	0.057	0.005
**(B) The best 10 individual models**			
**Ranking by PP**	**Model**	**PP**	$\hat{\theta }$ **_λ_**
1	Iron	0.725	0.344
3	TfSat	0.117	0.217
1, 2	Iron, Ferritin	0.042	0.313, 0.069
1, 3	Iron, TfSat	0.038	0.338, 0.004
1, 4	Iron, TIBC	0.036	0.448, 0.007
3, 4	TfSat, TIBC	0.016	0.393, 0.182
2, 3	Ferritin, TfSat	0.008	0.104, 0.186
4	TIBC	0.006	−0.215
1, 3, 4	Iron, TfSat, TIBC	0.004	0.309, 0.175, 0.159
1, 2, 4	Iron, Ferritin, TIBC	0.002	0.421, 0.079, 0.076

Results were generated using the MR-BMA approach. In total, 4 measured systemic iron statuses genetically instrumented by 6 SNVs were assessed as risk factors. All of the risk factors and the best ten individual models are presented. A positive causal estimate ($\hat{\theta }$_MACE_ or $\hat{\theta }$_λ_) indicates a risk factor, whereas a negative value indicates a protective effect as suggested by the model. $\hat{\theta }$_MACE_ is the model-averaged causal effect of a risk factor and $\hat{\theta }$_λ_ is the causal effect estimate for a specific model. Abbreviations: MIP, marginal inclusion probability; MR, Mendelian randomization; MR-BMA, MR based on Bayesian model averaging; PP, posterior probability; SNV, single-nucleotide variant; TIBC, total iron-binding capacity; TfSat, transferrin saturation.

**Table 3:** Ranking of risk factors and models (sets of risk factors) for hepatic fibrosis/cirrhosis.

**(A) Model averaging for risk factors**			
**Ranking by MIP**	**Risk factor**	**MIP**	$\hat{\theta }$ **_MACE_**
1	TIBC	0.604	−0.240
2	TfSat	0.235	0.051
3	Iron	0.212	0.049
4	Ferritin	0.125	0.057
**(B) The best 10 individual models**			
**Ranking by PP**	**Model**	**PP**	$\hat{\theta }$ **_λ_**
4	TIBC	0.476	−0.358
3	TfSat	0.162	0.330
1	Iron	0.132	0.493
2	Ferritin	0.066	0.825
1, 4	Iron, TIBC	0.047	−0.331, −0.569
3, 4	TfSat, TIBC	0.040	−0.271, −0.632
2, 4	Ferritin, TIBC	0.031	−0.018, −0.363
1, 3	Iron, TfSat	0.015	−0.137, 0.416
2, 3	Ferritin, TfSat	0.011	0.103, 0.300
1, 2	Iron, Ferritin	0.01	0.400, 0.205

Results were generated using the MR-BMA approach. In total, 4 measured circulating lipid traits genetically instrumented by 6 SNVs were assessed as risk factors. All of the risk factors and the best ten individual models are presented. A positive causal estimate ($\hat{\theta }$_MACE_ or $\hat{\theta }$_λ_) indicates a risk factor, whereas a negative value indicates a protective effect as suggested by the model. $\hat{\theta }$_MACE_ is the model-averaged causal effect of a risk factor and $\hat{\theta }$_λ_ is the causal effect estimate for a specific model. Abbreviations: MIP, marginal inclusion probability; MR, Mendelian randomization; MR-BMA, MR based on Bayesian model averaging; PP, posterior probability; SNV, single-nucleotide variant; TIBC, total iron-binding capacity; TfSat, transferrin saturation.

## Data Availability

The original contributions presented in this study are included in the article or in the data repositories listed in the References. Further inquiries can be directed to the corresponding authors.

## Supplementary Materials

The following supporting information can be downloaded at https://www.mdpi.com/article/10.3390/metabo16060356/s1. Figure S1: Causal effects of iron statues on hepatic steatosis by univariable MR analysis; Figure S2: Causal effects of iron statues on hepatic fibrosis/cirrhosis by univariable MR analysis; Table S1: GWAS data sources and cohort characteristics of systemic iron status and MASLD; Table S2: Characteristics of genetic instruments used in the strategy 1; Table S3: Characteristics of genetic instruments used in the strategy 2; Table S4: Characteristics of genetic instruments used in the strategy 3; Table S5: The R^2^ and F of SNVs used in the strategy 1 in univariable Mendelian randomization analyses; Table S6: The R^2^ and F of SNVs used in the strategy 2 in univariable Mendelian randomization analyses; Table S7: The R^2^ and F of SNVs used in the strategy 3 in univariable Mendelian randomization analyses; Table S8: The power of systemic iron status in Hepatic steatosis in univariable Mendelian randomization analyses; Table S9: The power of systemic iron status in Hepatic fibrosis/cirrhosis in univariable Mendelian randomization analyses; Table S10: GWAS Catalog cross-references for instrument SNVs used in the strategy 1; Table S11: GWAS Catalog cross-references for instrument SNVs used in the strategy 2; Table S12: GWAS Catalog cross-references for instrument SNVs used in the strategy 3; Table S13: Pleiotropy used in the strategy 1 of Hepatic steatosis; Table S14: Pleiotropy used in the strategy 1 of Hepatic fibrosis/cirrhosis; Table S15: Heterogeneity used in the strategy 1 of Hepatic steatosis; Table S16: Heterogeneity used in the strategy 1 of Hepatic fibrosis/cirrhosis; Table S17: Association between genetically instrumented systemic iron status and Hepatic steatosis; Table S18: Association between genetically instrumented systemic iron status and Hepatic fibrosis/cirrhosis; Table S19: Model diagnostics for MR-BMA analysis using q statistic for the instruments based on the best individual models with PP > 0.02 used in the strategy 1 of Hepatic steatosis; Table S20: Model diagnostics for MR-BMA analysis using q statistic for the instruments based on the best individual models with PP > 0.02 used in the strategy 1 of Hepatic fibrosis/cirrhosis; Table S21: Model diagnostics for MR-BMA analysis using Cd for the instruments based on the best individual models with PP > 0.02 used in the strategy 1 of Hepatic steatosis; Table S22: Model diagnostics for MR-BMA analysis using Cd for the instruments based on the best individual models with PP > 0.02 used in the strategy 1 of Hepatic fibrosis/cirrhosis. Supplementary File: STROBE-MR checklist.

## Abbreviations

95% CI, 95% confidence interval; ACSL4, acyl-CoA synthetase long-chain family member 4; CAD, coronary artery disease; FEV1, forced expiratory volume in 1 s; FVC, forced vital capacity; GWAS, genome-wide association study; HCC, hepatocellular carcinoma; HDAC3, histone deacetylase 3; ICD, the International Classification of Diseases; IV, instrumental variable; IVW, inverse-variance weighting; LD, linkage disequilibrium; MIP, marginal inclusion probability; MASH, metabolic dysfunction-associated steatohepatitis; MASLD, metabolic dysfunction-associated steatotic liver disease; MR, Mendelian randomization; MR-BMA, Mendelian randomization based on Bayesian model averaging; MRMix, Mendelian randomization analysis using mixture model; MACE, model-averaged causal estimate; OR, odds ratio; PP, posterior probability; ROS, reactive oxygen species; SNV, single-nucleotide variation; SLC7A11, solute carrier family 7 member 11; SD, standard deviation; TIBC, total iron-binding capacity; TfSat, transferrin saturation; T2DM, type 2 diabetes mellitus; θ, the estimates of causal effects generated by MRMix approach; π_0_, the proportion of valid instrumental variables; σ^2^, the unknown variance parameter associated with the invalid instrumental variables.
