# Supplementary material for: Mendelian Randomization Analysis of Systemic Iron Status and Risk of Metabolic Dysfunction-Associated Steatotic Liver Disease
Source: Metabolites. 2026 May 25;16(6):356. doi: 10.3390/metabo16060356 (PMC13302985; doi:10.3390/metabo16060356)
Supplement: Supplementary file 1 [file metabolites-16-00356-s001.zip › STROBE-MR-checklist-fillable.pdf]

STROBE-MR checklist of recommended items to address in reports of Mendelian randomization studies[1 2]

Item No.	Section	Checklist item	Page No.	Relevant text from manuscript
1	**TITLE and ABSTRACT**	Indicate Mendelian randomization (MR) as the study's design in the title and/or the abstract if that is a main purpose of the study	1-5	Title: "Mendelian Randomization Analysis of Systemic Iron Status and Risk of Metabolic Dysfunction-Associated Steatotic Liver Disease"<br>Abstract: "This study investigated the causal role of systemic iron status in MASLD progression."
	**INTRODUCTION**			
2	**Background**	Explain the scientific background and rationale for the reported study. What is the exposure? Is a potential causal relationship between exposure and outcome plausible? Justify why MR is a helpful method to address the study question	5-8<br>Figure 1	"Iron plays a crucial catalytic role in the Haber–Weiss reaction. Free ferrous iron promotes the conversion of superoxide anions and hydrogen peroxide into highly reactive hydroxyl radicals. Excessive hydroxyl radicals trigger intense oxidative stress, promote lipid peroxidation, and induce hepatocyte damage and ferroptosis. Therefore, this metal-catalyzed reaction represents an important mechanistic link between iron overload, oxidative stress, and the progression of MASLD and hepatic fibrosis [9]. Ferroptosis is an iron-dependent form of regulated cell death driven by the catastrophic accumulation of lipid peroxides on cellular membranes. It is primarily caused by the failure of the glutathione peroxidase 4 (GPX4)-dependent antioxidant system, which normally detoxifies phospholipid hydroperoxides [10]. In the context of MASLD and MASH, ferroptosis serves as a critical driver facilitating the transition from simple steatosis to metabolic dysfunction-associated steatohepatitis and progressive hepatic fibrosis [11]. Therefore, therapeutic strategies targeting ferroptosis—such as iron chelation, GPX4 activation, or antioxidant supplementation—hold promise for halting disease progression. Experimental studies consistently show that MASLD models exhibit hepatic iron accumulation and lipid peroxidation, along with dysregulation of ferroptosis-related pathways, supporting their role in disease pathogenesis [12]."

				"To address these knowledge gaps, we performed a comprehensive two-sample MR analysis to systematically dissect the causal impact of four core iron status biomarkers (serum iron, ferritin, TfSat, and TIBC) on MASLD progression (Figure 1A). By integrating large-scale GWAS summary statistics from FinnGen and UK Biobank (encompassing 8,785 hepatic steatosis cases/912,105 controls and 3,798 hepatic fibrosis/cirrhosis cases/904,599 controls of European ancestry) [21], employing inverse-variance weighted (IVW), weighted median, MR-Egger regression, MR-PRESSO, and heterogeneity tests, and implementing three rigorous instrumental variable (IV) selection strategies (Figure 1B)."
3	**Objectives**	State specific objectives clearly, including pre-specified causal hypotheses (if any). State that MR is a method that, under specific assumptions, intends to estimate causal effects	8	"In this study, our objective was to: (1) evaluate the causal role of systemic iron status in hepatic steatosis and fibrosis progression; (2) identify stage-specific iron biomarkers for MASLD risk stratification; (3) provide robust genetic evidence to support iron homeostasis as a rational therapeutic target for MASLD prevention and clinical management. This study advances the field by resolving ambiguities from prior observational and limited MR studies, offering robust causal evidence into the role of iron metabolism in MASLD pathogenesis."
	**METHODS**			
4	**Study design and data sources**	Present key elements of the study design early in the article. Consider including a table listing sources of data for all phases of the study. For each data source contributing to the analysis, describe the following:		
	a)	Setting: Describe the study design and the underlying population, if possible. Describe the setting, locations, and relevant dates, including periods of recruitment, exposure, follow-up, and data collection, when available.	Supplementary Table S1	
	b)	Participants: Give the eligibility criteria, and the sources and methods of selection of participants. Report the sample size, and whether any power or sample size calculations were carried out prior to the main analysis	9<br>Supplementary Table S1	"Summary statistics for systemic iron status were obtained from a meta-analysis of three genome-wide association studies conducted in the UK, Iceland, and Denmark. These studies reported genetic associations with four iron-related biomarkers: serum iron (n = 163,511), ferritin (n = 246,139), transferrin saturation (TfSat; n = 131,471), and total iron-binding

			capacity (TIBC; n = 135,430) [22]. Detailed cohort characteristics are provided in Supplementary Table S1."<br>"Genome-wide association summary statistics for hepatic steatosis and hepatic fibrosis/cirrhosis were obtained from large biobank resources including the FinnGen and UK Biobank databases. The hepatic steatosis dataset included 8,785 cases of European ancestry and 912,105 controls, while the hepatic fibrosis/cirrhosis dataset contained 3,798 cases and 904,599 controls of European ancestry [21]. Disease definitions were based on ICD-10 diagnostic codes: hepatic steatosis (ICD-10 K76.0) and hepatic fibrosis/cirrhosis (ICD-10 K74), individuals with excessive or long-term alcohol intake were excluded during participant enrollment, detailed information is available in the references."
c)	Describe measurement, quality control and selection of genetic variants	10	"A series of procedures for quality control was performed to identify valid instrumental SNVs. First, variants significantly associated with iron-related exposures at the genome-wide threshold ($p < 5 \times 10^{-8}$) were selected as candidate IVs. When the selected SNVs were not present in the outcome datasets, proxy variants were identified based on linkage disequilibrium utilizing the 1000 Genomes Project's European reference panel ($r^2 > 0.8$)."
d)	For each exposure, outcome, and other relevant variables, describe methods of assessment and diagnostic criteria for diseases	9	"Disease definitions were based on ICD-10 diagnostic codes: hepatic steatosis (ICD-10 K76.0) and hepatic fibrosis/cirrhosis (ICD-10 K74), individuals with excessive or long-term alcohol intake were excluded during participant enrollment, detailed information is available in the references. Each release's FinnGen disease end point definitions and corresponding controls may be found at https://www.finngen.fi/en/researchers/clinical-endpoints [21]."
e)	Provide details of ethics committee approval and participant informed consent, if relevant	9	"All datasets used in this study are publicly available, and the original studies received

					ethical approval with informed consent from participants.”
5	**Assumptions**		Explicitly state the three core IV assumptions for the main analysis (relevance, independence and exclusion restriction) as well assumptions for any additional or sensitivity analysis	9<br>Figure 1	“Genetic variants (single-nucleotide variants, SNVs) were used as IVs. Three fundamental presumptions underpin the MR framework’s validity: the genetic variants must have a strong correlation with the exposure; they must be unaffected by potential confounders that could affect the exposure–outcome relationship; and they must only influence the outcomes through the exposure, not through other pathways. Figure 1 shows a schematic overview of the study design.”
6	**Statistical methods: main analysis**		Describe statistical methods and statistics used		
		a)	Describe how quantitative variables were handled in the analyses (i.e., scale, units, model)	10-11	“The relationship between systemic iron levels and hepatic steatosis, as well as hepatic fibrosis/cirrhosis, was assessed through several MR techniques. Odds ratios (ORs) were determined for every one standard deviation rise in genetically inferred iron status. The main analysis employed the multiplicative random-effects IVW method, known for its ability to generate reliable estimates when multiple instrumental variables are used [25].”
		b)	Describe how genetic variants were handled in the analyses and, if applicable, how their weights were selected	11	“Weak instrument bias was thought to be indicated by an F-statistic less than 10. Lastly, Burgess’s design-based power calculation method was used to evaluate the statistical power of the Mendelian randomization analysis [31].”
		c)	Describe the MR estimator (e.g. two-stage least squares, Wald ratio) and related statistics. Detail the included covariates and, in case of two-sample MR, whether the same covariate set was used for adjustment in the two samples	11	“Additional MR methods were applied to assess the robustness of the findings, including fixed-effect IVW, weighted median [26], simple median, penalized weighted median, and MR-Egger regression [27]. To further account for and reduce potential bias induced by horizontal pleiotropy, a supplementary mixture-model-based Mendelian randomization method, which is MRMix, was applied in the present analysis [28].”

		d)	Explain how missing data were addressed	N/A	
		e)	If applicable, indicate how multiple testing was addressed	12	"Bonferroni correction was used to account for multiple testing, and the significance threshold was set at $p < 0.00625$ (0.05/8, corresponding to four exposures and two outcomes) was seen as statistically significant, while p-values between 0.00625 and 0.05 were thought to be suggestive."
7	**Assessment of assumptions**		Describe any methods or prior knowledge used to assess the assumptions or justify their validity	12	"Cochran's Q statistic was used to measure heterogeneity among IVs, and the intercept from MR-Egger regression was used to examine potential horizontal pleiotropy [32]."
8	**Sensitivity analyses and additional analyses**		Describe any sensitivity analyses or additional analyses performed (e.g. comparison of effect estimates from different approaches, independent replication, bias analytic techniques, validation of instruments, simulations)	12	"Cochran's Q statistic was used to measure heterogeneity among IVs, and the intercept from MR-Egger regression was used to examine potential horizontal pleiotropy [32]. Outlying or influential variants were identified using Cook's distance (Cd) and Cochran's Q statistic. SNVs that had a Cd value higher than the relevant F-distribution's median or a Q statistic larger than 10 were not included in the analysis [31]."<br>"Bonferroni correction was used to account for multiple testing, and the significance threshold was set at $p < 0.00625$ (0.05/8, corresponding to four exposures and two outcomes) was seen as statistically significant, while p-values between 0.00625 and 0.05 were thought to be suggestive."
9	**Software and pre-registration**				
		a)	Name statistical software and package(s), including version and settings used	12	"All analyses were conducted in R (version 4.5.1; R Foundation for Statistical Computing) using the Two Sample MR (version 0.6.22) and MRMix packages (version 0.1.0)."
		b)	State whether the study protocol and details were pre-registered (as well as when and where)	N/A	
	**RESULTS**				
10	**Descriptive data**				

		a)	Report the numbers of individuals at each stage of included studies and reasons for exclusion. Consider use of a flow diagram	N/A	
		b)	Report summary statistics for phenotypic exposure(s), outcome(s), and other relevant variables (e.g. means, SDs, proportions)	N/A	
		c)	If the data sources include meta-analyses of previous studies, provide the assessments of heterogeneity across these studies	N/A	
		d)	For two-sample MR:<br>i. Provide justification of the similarity of the genetic variant-exposure associations between the exposure and outcome samples<br>ii. Provide information on the number of individuals who overlap between the exposure and outcome studies	Supplementary Table S1	
11	**Main results**				
		a)	Report the associations between genetic variant and exposure, and between genetic variant and outcome, preferably on an interpretable scale	12-13<br>Supplementary Tables S1-S2,S5-S9	"Supplementary Table S1 listed the impact on iron levels for each copy of the instrument SNV effect allele, represented as the number of SD from the mean. Thus, after significance threshold screening, LD clumping, proxy selection, and exclusion of known pleiotropic variants, Strategy 1 yielded 19 independent SNVs that were used as the Tier 1 IV set (Supplementary Table S2). All employed IVs were sufficiently strong, with F-statistics exceeding 10 across Strategies 1, 2, and 3. The specific R2 values and F values are provided in Supplementary Tables S5-S7. According to post hoc power estimates, the study's sample size was adequate for both hepatic steatosis (Supplementary Table S8) and hepatic fibrosis/cirrhosis (Supplementary Table S9)."
		b)	Report MR estimates of the relationship between exposure and outcome, and the measures of uncertainty from the MR analysis, on an interpretable scale, such as odds ratio or relative risk per SD difference	13<br>Figure 2 and 3,<br>Supplementary Figures 1 and 2	"Overall, elevated iron, ferritin, and TfSat levels were associated with an elevated risk of the liver injury mediated by genetic susceptibility to higher iron status; this finding was further supported by lower TIBC. The directional consistency and significance of differences for 137 separate SNVs in hepatic steatosis were assessed in accordance with Tier 1. Iron (OR: 1.42; 95% CI: 1.34, 1.50; $p < 0.00625$), ferritin (OR: 1.84; 95% CI: 1.55, 2.18; $p < 0.00625$), and transferrin saturation (TfSat; OR: 1.24; 95% CI: 1.19, 1.30; $p < 0.00625$) all showed a

					significantly elevated systemic iron status. On the other hand, total iron-binding capacity (TIBC; OR: 0.81; 95% CI: 0.77, 0.85; $p < 0.00625$) showed that individuals with lower systemic iron status exhibited a decreased risk (Figure 2, Supplementary Figure 1).”<br>“The findings showed a substantial causal relationship between an increased risk of hepatic fibrosis/cirrhosis and all four measures of elevated systemic iron status : iron (OR: 1.66; 95% CI: 1.29, 2.14; $p < 0.00625$), ferritin (OR: 2.52; 95% CI: 1.52, 4.18; $p < 0.00625$), TfSat (OR:1.40; 95% CI: 1.19, 1.63; $p < 0.00625$), and TIBC (OR: 0.70; 95% CI: 0.60, 0.81; $p < 0.00625$). Our findings support that increased systemic iron status is causally associated with a higher risk of hepatic steatosis and progression to hepatic fibrosis/cirrhosis. (Figure 3, Supplementary Figure 2).”
		c)	If relevant, consider translating estimates of relative risk into absolute risk for a meaningful time period	N/A	
		d)	Consider plots to visualize results (e.g. forest plot, scatterplot of associations between genetic variants and outcome versus between genetic variants and exposure)	Figure 2 and 3	
12	**Assessment of assumptions**				
		a)	Report the assessment of the validity of the assumptions	14<br>Supplementary Tables S13-S16	“Hepatic steatosis and hepatic fibrosis/cirrhosis did not exhibit horizontal pleiotropy in terms of the MR-Egger regression intercept ($p > 0.05$) (Supplementary Tables S13-S14). Lastly, in hepatic steatosis and hepatic fibrosis/cirrhosis, the Cochran's Q statistics demonstrated that there was no heterogeneity was detected between the assessed IV values for each biomarker when using the MR-Egger and IVW approaches (Supplementary Tables S15-S16).”
		b)	Report any additional statistics (e.g., assessments of heterogeneity across genetic variants, such as I^2, Q statistic or E-value)	16<br>Table 2 and 3	“Additionally, the non-linear MR-BMA method was utilized with IVs chosen by Tier 1 to rank the best models for hepatic steatosis and hepatic fibrosis/cirrhosis. The highest-ranked iron biomarkers for hepatic steatosis were iron (MIP: 0.85; θMACE: 0.295), TfSat (MIP: 0.187; θMACE: 0.035), ferritin (MIP: 0.057; θMACE:

					0.005), and TIBC (MIP: 0.067; θMACE: 0.005). These biomarkers were also used in the best models for PP > 0.02 (Table 2). The highest-ranked iron biomarkers for hepatic fibrosis/cirrhosis were TfSat (MIP: 0.235; θMACE: 0.051), iron (MIP: 0.212; θMACE: 0.049), ferritin (MIP: 0.125; θMACE: 0.057), and TIBC (MIP: 0.604; θMACE: -0.24) (Table 3)."
13	**Sensitivity analyses and additional analyses**				
		a)	Report any sensitivity analyses to assess the robustness of the main results to violations of the assumptions	14<br>Supplementary Tables S10-S16	"This study used the gwasrapidd package in R to programmatically screen each SNV in the GWAS Catalog in order to reduce horizontal pleiotropy. In 29th November 2025, the catalog queries were executed. Substantial genome-wide associations were identified in domains that may represent alternative pathways contributing to the risk of hepatic fibrosis/cirrhosis and hepatic steatosis. The associated rsIDs variants detected by this screening — were merged into an exclusion list and removed from the MR analysis set. Additionally, a variable was eliminated prior to MR estimation if a SNV showed genome-wide significance for the outcomes including hepatic steatosis and hepatic fibrosis/cirrhosis. For the remaining instruments, this study kept the catalog notes to help with findings interpretation. To uncover potential alternative biological pathways and support variant filtering, prior genome-wide significant associations for each SNV and eligible proxy were retrieved from the GWAS Catalog; comprehensive cross-references are included in Supplementary Tables S10-S12. Hepatic steatosis and hepatic fibrosis/cirrhosis did not exhibit horizontal pleiotropy in terms of the MR-Egger regression intercept (p > 0.05) (Supplementary Tables S13-S14). Lastly, in hepatic steatosis and hepatic fibrosis/cirrhosis, the Cochran's Q statistics demonstrated that there was no heterogeneity was detected between the assessed IV values for each biomarker when using the MR-Egger

				and IVW approaches(Supplementary Tables S15-S16).”
		b) Report results from other sensitivity analyses or additional analyses	Supplementary Tables S13-S18	
		c) Report any assessment of direction of causal relationship (e.g., bidirectional MR)	N/A	
		d) When relevant, report and compare with estimates from non-MR analyses	N/A	
		e) Consider additional plots to visualize results (e.g., leave-one-out analyses)	N/A	
	**DISCUSSION**			
14	**Key results**	Summarize key results with reference to study objectives	16-23	“In this large-scale MR study involving populations from Finland and the UK, a two-sample MR design was employed to investigate the role of genetically regulated iron metabolism in genetic modulation of iron metabolism in the risk of hepatic steatosis and hepatic fibrosis/cirrhosis. Our findings showed key findings revealed strong associations between genetically predicted hepatic iron levels and an elevated risk of hepatic steatosis. Additionally, across multiple datasets, genetically predicted MASLD and MASH were robustly associated with higher levels of serum ferritin, iron, and TfSat levels.”<br>“Systemic iron homeostasis plays a key role in the pathogenesis of metabolic liver diseases, and our MR study provides genetic evidence supporting a causal role of elevated systemic iron status in both hepatic steatosis and hepatic fibrosis/cirrhosis. These findings align with and extend prior epidemiological and experimental evidence, while addressing critical limitations of observational research through genetic instrumental variable analysis.”
15	**Limitations**	Discuss limitations of the study, taking into account the validity of the IV assumptions, other sources of potential bias, and imprecision. Discuss both direction and magnitude of any potential bias and any efforts to address them	22-23	“Nevertheless, our study also has several limitations. First, the analysis was based solely on summary-level GWAS data, which prevented stratified analyses by factors such as age, sex, or drinking status, potentially hiding variations across different subgroups. Second, the exposure–response relationship between iron

				homeostasis and the severity of hepatic steatosis or hepatic fibrosis/cirrhosis—such as the degree of hepatic steatosis or the stage of hepatic fibrosis/cirrhosissuch as the level of hepatic steatosis in hepatic steatosis or the stage of hepatic fibrosis/cirrhosis—was not examined; the study only focused on the presence or absence of disease risk, not the severity of that risk.”
16	**Interpretation**			
		a) Meaning: Give a cautious overall interpretation of results in the context of their limitations and in comparison with other studies	23	“In conclusion, this rigorously designed two-sample Mendelian randomization study provides strong genetic evidence that systemic iron homeostasis dysregulation is a causal driver of MASLD and MASH development and progression, with ferroptosis as a key mechanistic mediator linking iron overload to hepatocellular injury and fibrogenesis. Through systematic interrogation of four core iron biomarkers (serum iron, ferritin, TfSat, and TIBC) across the disease continuum from early hepatic steatosis to advanced fibrosis/cirrhosis, we leveraged GWAS encompassing 8,785 steatosis cases with 912,105 controls and 3,798 fibrosis/cirrhosis cases with 904,599 controls. Integration of three instrumental variable selection strategies, multiple robust MR and comprehensive sensitivity analyses confirmed consistent causal associations between elevated serum iron, ferritin, and TfSat, decreased TIBC, and an increased risk of hepatic steatosis, with all associations achieving statistical significance at $p < 0.00625$ after Bonferroni correction. MR-BMA prioritized iron and ferritin as top causal factors, with ferritin exerting the most pronounced effect on advanced fibrosis/cirrhosis (OR: 2.52, 95% CI: 1.52, 4.18, $p < 0.00625$). These findings resolve longstanding ambiguities from observational studies, identify stage-specific biomarkers for risk stratification and establish iron homeostasis and ferroptosis as synergistic rational therapeutic targets, offering critical insights to inform precision prevention and clinical management strategies for mitigating the global public health burden of metabolic dysfunction-

			associated steatotic liver disease. In summary, our findings highlight the interplay between systemic iron status and ferroptosis in the pathogenesis of MASLD and MASH.”
b)	Mechanism: Discuss underlying biological mechanisms that could drive a potential causal relationship between the investigated exposure and the outcome, and whether the gene-environment equivalence assumption is reasonable. Use causal language carefully, clarifying that IV estimates may provide causal effects only under certain assumptions	19-20	“Mechanistically, excess iron promotes the generation of reactive oxygen species (ROS) and lipid peroxidation. Iron overload can activate hepatic stellate cells through oxidative stress and inflammatory signaling. This activation promotes collagen synthesis and fibrogenic pathways, which contribute to the development of the liver fibrosis [47,48]. Ferroptosis, an iron-dependent type of controlled cell death that exacerbates inflammation and hepatocellular damage, can be triggered by this process [49]. In mouse models of MASLD, special diets induce hepatic iron accumulation, lipid peroxidation, and downregulation of ferroptosis-related genes, directly linking iron dysregulation to steatotic liver disease pathogenesis [50].”
c)	Clinical relevance: Discuss whether the results have clinical or public policy relevance, and to what extent they inform effect sizes of possible interventions	21	“In clinical practice, our MR analyses help mitigateeliminate confounding biases and provide genetic evidence for the association between iron status and liver disease, offering up-to-date and comprehensive insights into their causal relationship. These findings underscore the robustness and reliability of our results, providing strong support for causal inference. Elevated systemic iron status was associated with an increased risk of both hepatic steatosis and hepatic fibrosis/cirrhosis, although the direction and statistical significance of these associations varied across different iron biomarkers. Specifically, for hepatic steatosis, higher levels of serum iron, ferritin, and TfSat were significantly associated with an elevated disease risk, whereas a reduced TIBC was also associated with a significantly increased risk of hepatic steatosis. Regarding hepatic fibrosis/cirrhosis, elevated serum iron and TfSat levels showed a tendency toward increased risk, while decreased TIBC was significantly associated with a higher risk of hepatic fibrosis/cirrhosis.”

17	**Generalizability**	Discuss the generalizability of the study results (a) to other populations, (b) across other exposure periods/timings, and (c) across other levels of exposure	23	"Our future studies will be conducted in larger populations. Meanwhile, clinical intervention studies will be performed in patients with hepatic fibrosis and cirrhosis, using TfSat and ferritin as stage-specific targets."
	**OTHER INFORMATION**			
18	**Funding**	Describe sources of funding and the role of funders in the present study and, if applicable, sources of funding for the databases and original study or studies on which the present study is based	24	"This study was supported by research grants from the National Natural Science Foundation of China (32271214 to X.W.; 32330047 to F.W.; and 31970689 to J.M.) and the Starry Night Science Fund of Zhejiang University Shanghai Institute for Advanced Study (No. SN-ZJU-SIAS-0020)."
19	**Data and data sharing**	Provide the data used to perform all analyses or report where and how the data can be accessed, and reference these sources in the article. Provide the statistical code needed to reproduce the results in the article, or report whether the code is publicly accessible and if so, where	Supplementary Table S1	
20	**Conflicts of Interest**	All authors should declare all potential conflicts of interest	24	"The authors declare no competing interests."

This checklist is copyrighted by the Equator Network under the Creative Commons Attribution 3.0 Unported (CC BY 3.0) license.

1. Skrivankova VW, Richmond RC, Woolf BAR, Yarmolinsky J, Davies NM, Swanson SA, et al. Strengthening the Reporting of Observational Studies in Epidemiology using Mendelian Randomization (STROBE-MR) Statement. JAMA. 2021;under review.

2. Skrivankova VW, Richmond RC, Woolf BAR, Davies NM, Swanson SA, VanderWeele TJ, et al. Strengthening the Reporting of Observational Studies in Epidemiology using Mendelian Randomisation (STROBE-MR): Explanation and Elaboration. BMJ. 2021;375:n2233.
