# Supplementary material for: Mendelian Randomization Analysis of Systemic Iron Status and Risk of Metabolic Dysfunction-Associated Steatotic Liver Disease
Source: Metabolites. 2026 May 25;16(6):356. doi: 10.3390/metabo16060356 (PMC13302985; doi:10.3390/metabo16060356)
Supplement: Supplementary file 1 [file metabolites-16-00356-s001.zip › Supplementary Figures.pdf]

Mendelian Randomization Analysis of Systemic Iron Status and Risk of Metabolic Dysfunction-Associated Steatotic Liver Disease

Wuyang Yue,[1,2#] Yi Yang,[2#] Jinling Ma,[2] Jiale Zhang,[2] Xinhui Wang,[2] Junxia Min,[3*] and Fudi Wang[1,4*]*

1 The Second Affiliated Hospital, School of Public Health, State Key Laboratory of Experimental Hematology, Zhejiang University School of Medicine, Hangzhou 310058, China

2 School of Public Health, Sir Run Run Shaw Hospital, Zhejiang University School of Medicine, Hangzhou 310058, China

3 The First Affiliated Hospital, Institute of Translational Medicine, Zhejiang Key Laboratory of Frontier Medical Research on Cancer Metabolism, Zhejiang University School of Medicine, Hangzhou, 310058, China

4 Global Innovation Institute of Element Science (GIIES-JLU), The First Hospital of Jilin University, Changchun 130021, China

***Address correspondence to:**

Correspondence to: Xinhui Wang (Email: xinhuiwang@zju.edu.cn), Junxia Min (Email: junxiamin@zju.edu.cn), or Fudi Wang (Email: fwang@zju.edu.cn).

The file includes:

Supplementary Figures 1-2

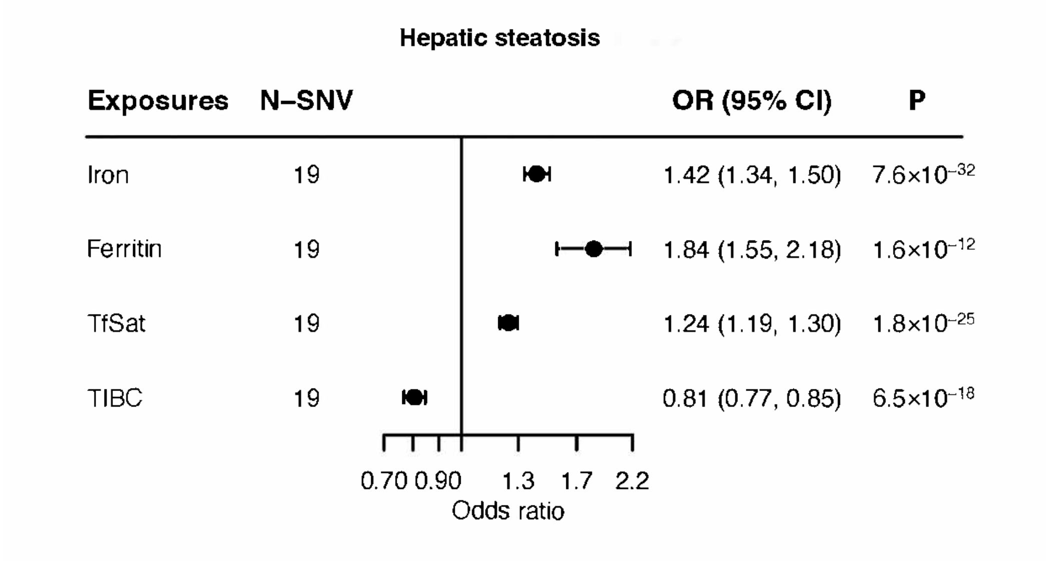

Supplementary Figure S1 Causal effects of iron statues on hepatic steatosis by univariable MR analysis. The estimated ORs reflect the impact of one SD increase in iron statues on hepatic steatosis, as determined through multiplicative random-effect inverse-variance weighted analysis. Abbreviations: 95% CI, 95% confidence interval; IVW, inverse-variance weighted; MR, Mendelian randomization; OR, odds ratio; SNV, single-nucleotide variation; SD, standard deviation; TIBC, total iron-binding capacity; TfSat, transferrin saturation.

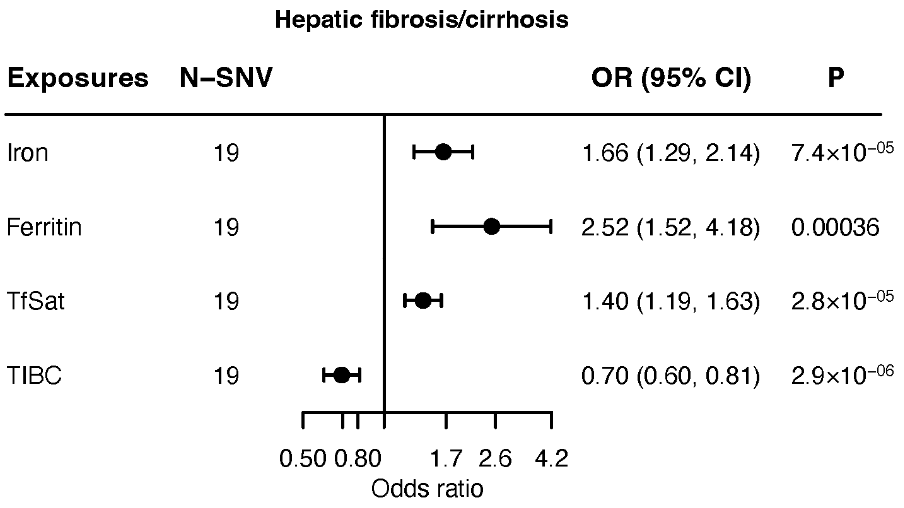

Supplementary Figure S2 Causal effects of iron statues on hepatic fibrosis/cirrhosis by univariable MR analysis. The estimated ORs reflect the impact of one SD increase in iron statues on hepatic fibrosis/cirrhosis, as determined through multiplicative random-effect inverse-variance weighted analysis. Abbreviations: 95% CI, 95% confidence interval; IVW, inverse-variance weighted; MR, Mendelian randomization; OR, odds ratio; SNV, single-nucleotide variation; SD, standard deviation; TIBC, total iron-binding capacity; TfSat, transferrin saturation.
